# Spontaneous activity of rat pretectal nuclear complex neurons *in vitro*

**DOI:** 10.1186/1471-2202-5-29

**Published:** 2004-08-27

**Authors:** Nora Prochnow, Matthias Schmidt

**Affiliations:** 1 Allgemeine Zoologie & Neurobiologie, Ruhr-Universität Bochum, 44780 Bochum, Germany

## Abstract

**Background:**

Neurons in the mammalian pretectum are involved in the control of various visual and oculomotor tasks. Because functionally independent pretectal cell populations show a wide variation of response types to visual stimulation *in vivo*, they may also differ in their intrinsic properties when recorded *in vitro*. We therefore performed whole-cell patch clamp recordings from neurons in the caudal third of the pretectal nuclear complex in frontal brain slices obtained from 3 to 6 week old hooded rats and tried to classify pretectal neurons electrophysiologically.

**Results:**

Pretectal neurons showed various response types to intracellular depolarizations, including bursting and regular firing behavior. One population of pretectal nuclear complex neurons could be particularly distinguished from others because they displayed spontaneous activity *in vitro*. These cells had more positive resting potentials and higher input resistances than cells that were not spontaneously active. The maintained firing of spontaneously active pretectal cells was characterized by only small variances in interspike intervals and thus showed a regular temporal patterning. The firing rate was directly correlated to the membrane potential. Removing excitatory inputs by blockade of AMPA and/or NMDA receptors did not change the spontaneous activity. Simultaneous blockade of excitatory and inhibitory synaptic input by a substitution of extracellular calcium with cobalt neither changed the firing rate nor its temporal patterning. Each action potential was preceeded by a depolarizing inward current which was insensitive to calcium removal but which disappeared in the presence of tetrodotoxin.

**Conclusions:**

Our results indicate that a specific subpopulation of pretectal neurons is capable of generating maintained activity in the absence of any external synaptic input. This maintained activity depends on a sodium conductance and is independent from calcium currents.

## Background

Neurons in the mammalian pretectal nuclear complex (PNC) are involved in the control of various oculomotor reflexes, like the pupillary light reflex and the optokinetic reflex (OKR). Pupil constriction is controlled by neurons in the olivary pretectal nucleus that project bilaterally to the Edinger-Westphal nucleus [1-7]. Slow eye movements during OKR are generated by neurons in the nucleus of the optic tract (NOT) and in the adjacent dorsal terminal nucleus (DTN) of the accessory optic system (AOS) which project to the inferior olive (IO) and the nucleus prepositus hypoglossi [8-13]. In addition, PNC neurons carry signals related to saccadic eye movements to the dorsal lateral geniculate nucleus (LGNd) [14-16] and to the extrageniculate thalamus [17]. Other, reciprocal, projections connect the PNC to its contralateral counterpart, to the ipsilateral superior colliculus, and to the other AOS nuclei. The functions of those projections, however, are still under debate [18-23].

Each projection target receives input from an independent PNC neuronal population. Therefore, multiple retrograde tracing, e.g. from contralateral PNC and IO [24], or LGNd and Pulvinar [25], does not double label PNC cells. Furthermore, neurons with different projection targets show different response properties *in vivo*. Thus, neurons involved in the pupillary light reflex respond tonically to the overall retinal luminance [1,3,6,26]. OKR-related neurons are directionally selective in response to slow movements of large visual stimuli [8,27-33]. Finally, PNC neurons that project to thalamic visual centers only respond to fast moving visual stimuli without directional selectivity [15,17,25,34-36]. Furthermore, activity patterns of visual responses also differ significantly between PNC cell populations. Thus, saccade-related PNC neurons show short high frequency activity bursts, while luminance neurons or OKR-related neurons exhibit tonic activity at moderate firing levels. Although such differences to some extent directly reflect the response properties of specific input systems, different intracellular properties might enforce activity patterns provided by different input systems.

We therefore studied intrinsic properties of rat PNC cells *in vitro. *In particular*, *we describe a population of cells in the caudo-lateral PNC that is characterized by intrinsically generated spontaneous activity *in vitro*, which is an unusual property for neurons in a sensory relay structure.

## Results

**Figure 1:**
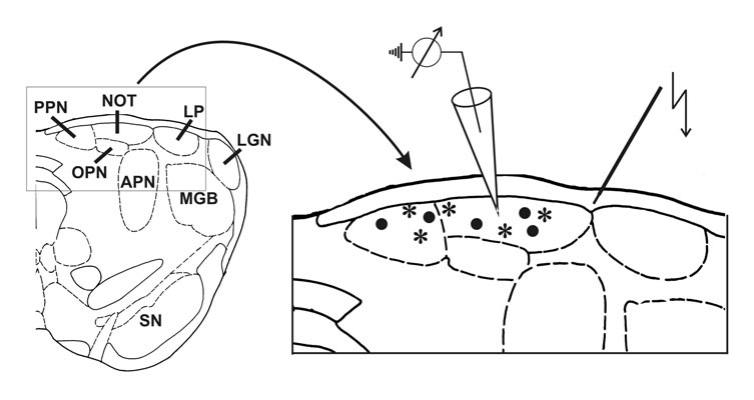
Schematic view of stimulation and recording sites. The box in the reconstructed section from the right midbrain in the left panel indicates the position of the PNC shown at higher magnification in the right panel (dorsal is up, medial is to the left). Neurons were recorded from the most dorsal and caudal nuclei of the PNC, the nucleus of the optic tract (NOT) and the posterior pretectal nucleus (PPN). No topographical segregation between spontaneously active cells (indicated by asterisks) and neurons without spontaneous activity (filled circles) was observed within the PNC. The stimulation electrode (black bar) was placed in the optic tract at the dorso-lateral border of the PNC medial to the lateral posterior thalamic nucleus (LP). Other abbreviations used, APN, anterior pretectal nucleus, OPN, olivary pretectal nucleus, LGN, lateral geniculate nucleus, MGB, medial geniculate body, SN, substantia nigra.

**Figure 2:**
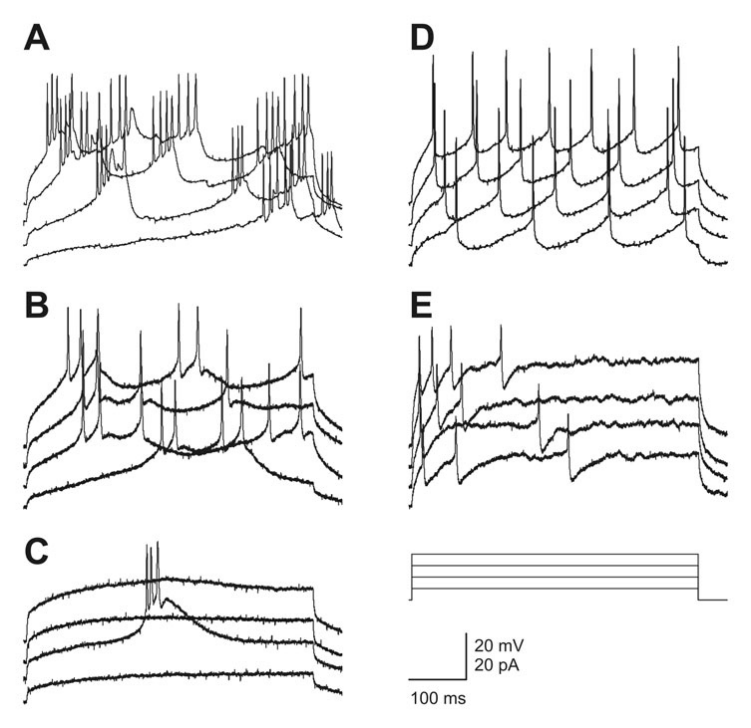
Different types of responses in PNC neurons evoked by depolarizing current injections. *A-C*, Whole-cell recordings from three individual PNC cells that showed burst firing in response to depolarizing currents. While the firing rate of cells in *A *and *B *monotonically increased with increasing currents, the cell in *C *only showed spikes in response to intermediate currents. *D*, Regular non-adapting spiking with monotonically increasing firing rates to increasing currents was observed in another population of cells. *E*, Responses from a PNC cell that showed irregular fast-adapting spiking. Although increasing currents in this cell decreased interspike intervals the total number of spikes elicited remained constant. Resting potentials of the cells were -61.6 mV, A, -62.5 mV, B, -61.2 mV, C, -58.4 mV, D, and -50.9 mV, E. Current amplitudes applied to each cell were 5, 10, 15, and 20 pA.

In total, we obtained whole-cell recordings from 114 pretectal nuclear complex (PNC) neurons. Slices included the caudal part of the pretectum (Fig. 1), cells were recorded from the NOT, the posterior pretectal nucleus (PPN), and the olivary pretectal nucleus (OPN). Depolarizing current injections induced various spike patterns, like bursting (Fig. 2A,2B,2C), non-adapting regular spiking (Fig. 2D), or irregular spiking (Fig. 2E). Usually, increasing the current amplitude also increased the firing rate, however, in about 31% (n = 35) of the cells, the firing rate showed a clear maximum in response to intermediate depolarizing current injections and decreased upon further depolarization (Fig. 2C). Input resistances ranged from 201.0 to 776.3 MΩ (mean 410 ± 166.5 MΩ), resting potentials varied between -41.0 and -74.3 mV (mean -54.6 ± 8.5 mV). All cells tested, irrespective of the response type, responded to OT stimulation.

### Characteristics of spontaneous activity

**Figure 3:**
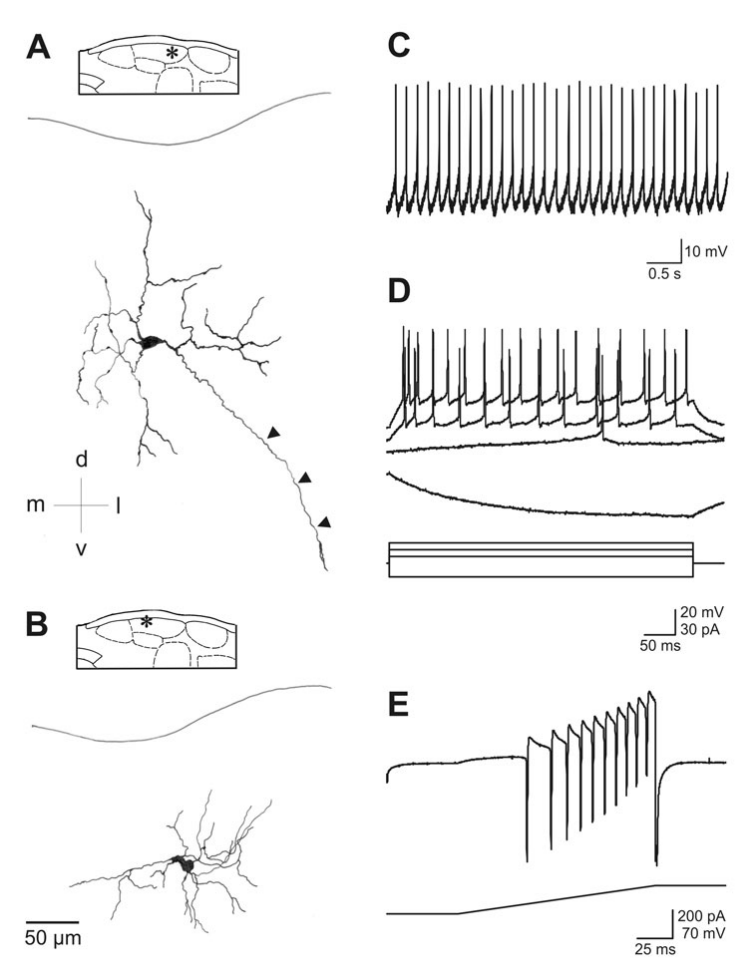
Morphological and physiological characteristics of PNC neurons with spontaneous activity *in vitro*. *A*, *B*, Reconstruction drawings of two biocytin-filled PNC neurons, insets indicate the cells' position within the PNC. Horizontal lines mark the dorsal border of the PNC, arrowheads in *A *point to the cell's axon. *C*, in the absence of injected currents, spontaneous regular spiking was observed in this neuron. *D*, Responses to intracellular current injection identify this cell as regular non-adapting. *E*, continuous depolarization starting at a membrane potential of -90 mV revealed this cell's spike threshold at -66.4 mV. d, dorsal, m, medial, v, ventral, l, lateral.

Of the cells recorded, 73 PNC neurons showed spontaneous firing at resting potential. Camera lucida reconstructions revealed that these cells were characterized by large fusiform cell bodies (diam. 15 μm and above) and multipolar dendritic trees that did not show any preference in their orientation. Whenever axons were also visible, they could be followed to leave the pretectal area in a ventro-lateral direction which indicates that these cells project to extrapretectal targets (arrowheads in Fig. 3A). Their dendritic morphologies, however, did not allow to distinguish spontaneously active cells from PNC neurons that were not spontaneously active.

Physiologically, all spontaneously active PNC neurons were characterized by a regular firing pattern when recorded at resting potential without any injected current (Fig. 3C). The firing rate at resting potential of spontaneously active PNC cells varied between 0.9 and 9.4 imp/s (mean 3.0 ± 2.1 imp/s). Depolarizing current injections induced tonic firing patterns with only marginal adaptation (Fig. 3D). Responses to hyperpolarizing current injections showed no sign of inward rectification. Furthermore, following cessation of hyperpolarizing current steps we never observed rebound spikes. Spontaneously active PNC cells on average had significantly higher input resistances (mean 454.1 ± 164.7 MΩ, p < 0.001), more positive resting potentials (mean -50.4 ± 7.0 mV, p < 0.001) and lower spike thresholds (mean -55.0 ± 3.96 mV, p < 0.001) than cells that did not show spontaneous activity (331.44 ± 137.1 MΩ, -58.4 ± 8.0 mV, and -40.66 ± 6.44 mV, respectively).

**Figure 4:**
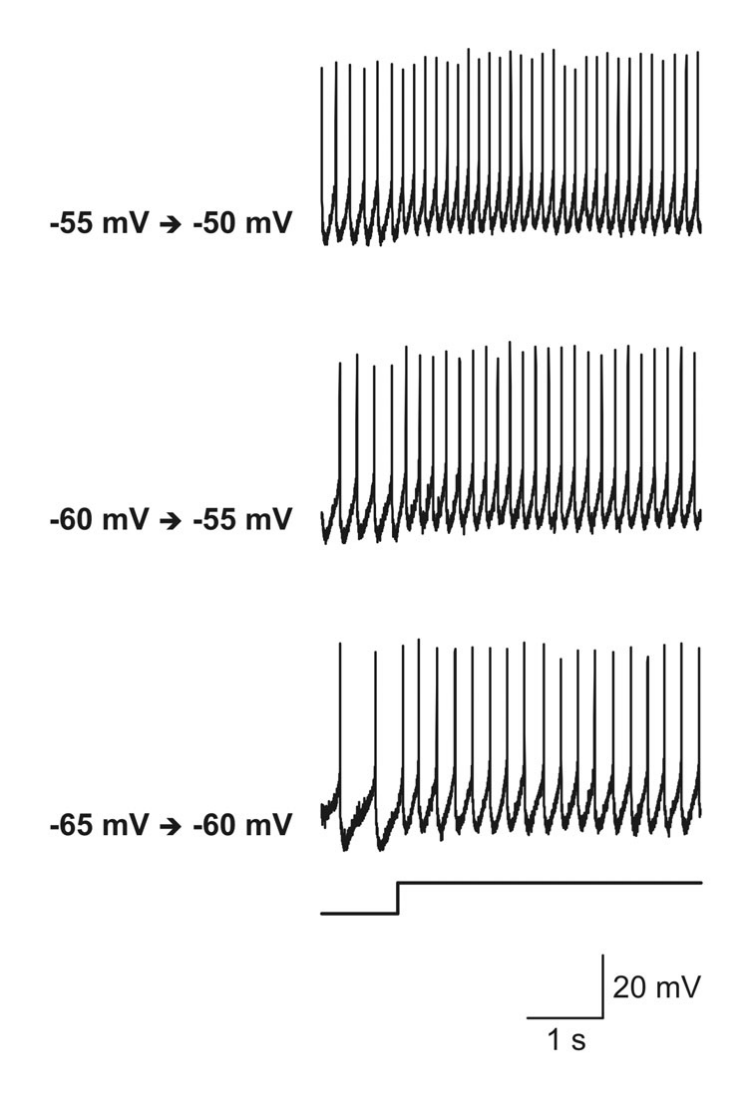
Response to intracellular depolarization of spontaneously active PNC cells. Depolarizing current steps induce tonic firing increases in this spontaneously active PNC cell. No phasic component appears in the response to the depolarization step. This behavior was a characteristic property of all spontaneously active PNC cells.

In order to characterize the spike adaptation behavior of spontaneously active PNC cells, the holding potentials were increased in 5 mV steps by appropriate current injections in all recorded cells. In response to these depolarization steps, cells showed tonic increases of their firing rate without any sign of firing rate adaptation (Fig. 4). Also, no phasic firing rate increases were observed following the depolarizations.

**Figure 5:**
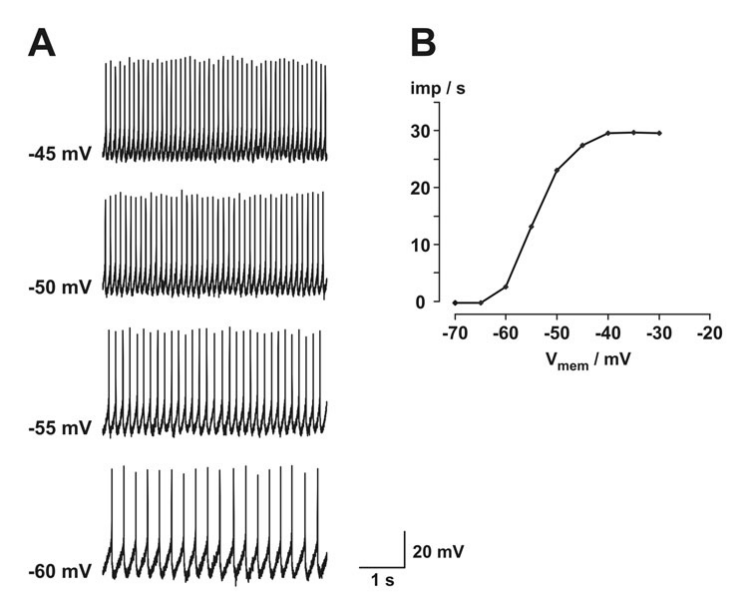
Correlation between firing rate and membrane potential in spontaneously active PNC cells. *A*, Incremental intracellular depolarization leads to increasing firing rates without changing the regular firing pattern. *B*, When the firing rate is plotted as a function of membrane potential a sinusoidal curve appears that frequently saturates at membrane potentials above -20 mV.

As could be already derived from current injections, the firing rate was directly correlated with the membrane potential. Increasing the membrane potential by positive current injections increased the firing rate until a maximum level was reached that could not be exceeded by further depolarization (Fig. 5A). Consequently, when the firing rate is plotted against the membrane potential, the course of the resulting function is sigmoidal (Fig. 5B).

**Figure 6:**
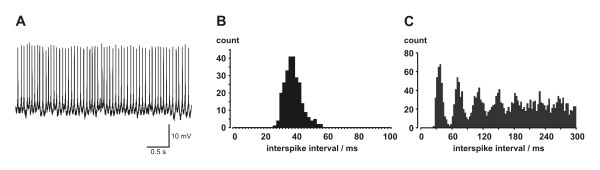
Regularity of the firing pattern of spontaneously active PNC cells. *A*, Current clamp recording from a PNC cell at slightly depolarized membrane potential. *B*, The interspike interval histogram shows a narrow Gaussian distribution with only little variation of interspike intervals. *C*, The regularity of the firing can also be derived from the autocorrelogram which exhibits multiple equally spaced peaks due to the little variation in the interspike intervals.

In order to get an impression about the regularity of the firing of spontaneously active PNC cells, interspike intervals (ISI) during maintained firing were analyzed in more detail (Fig. 6). Thus, maintained firing was recorded over a 10 s period at different membrane potentials and ISI histograms were generated from the recorded activity. ISIs obtained from these recordings followed a narrow unimodal Gaussian distribution with only little variation (Fig. 6B). According to the correlation between the firing rate and the membrane potential, depolarization of the cells resulted in shifts of the maximum of the Gaussian distribution towards lower ISI values. Depolarization, however, did not change the shape of the distribution. The regularity of the maintained firing of spontaneously active PNC cells is also supported by autocorrelograms of the recorded spike trains (Fig. 6C). The appearance of multiple equally spaced peaks in the autocorrelogram results from the regular timing of single spikes.

### Generation of spontaneous activity *in vitro*

**Figure 7:**
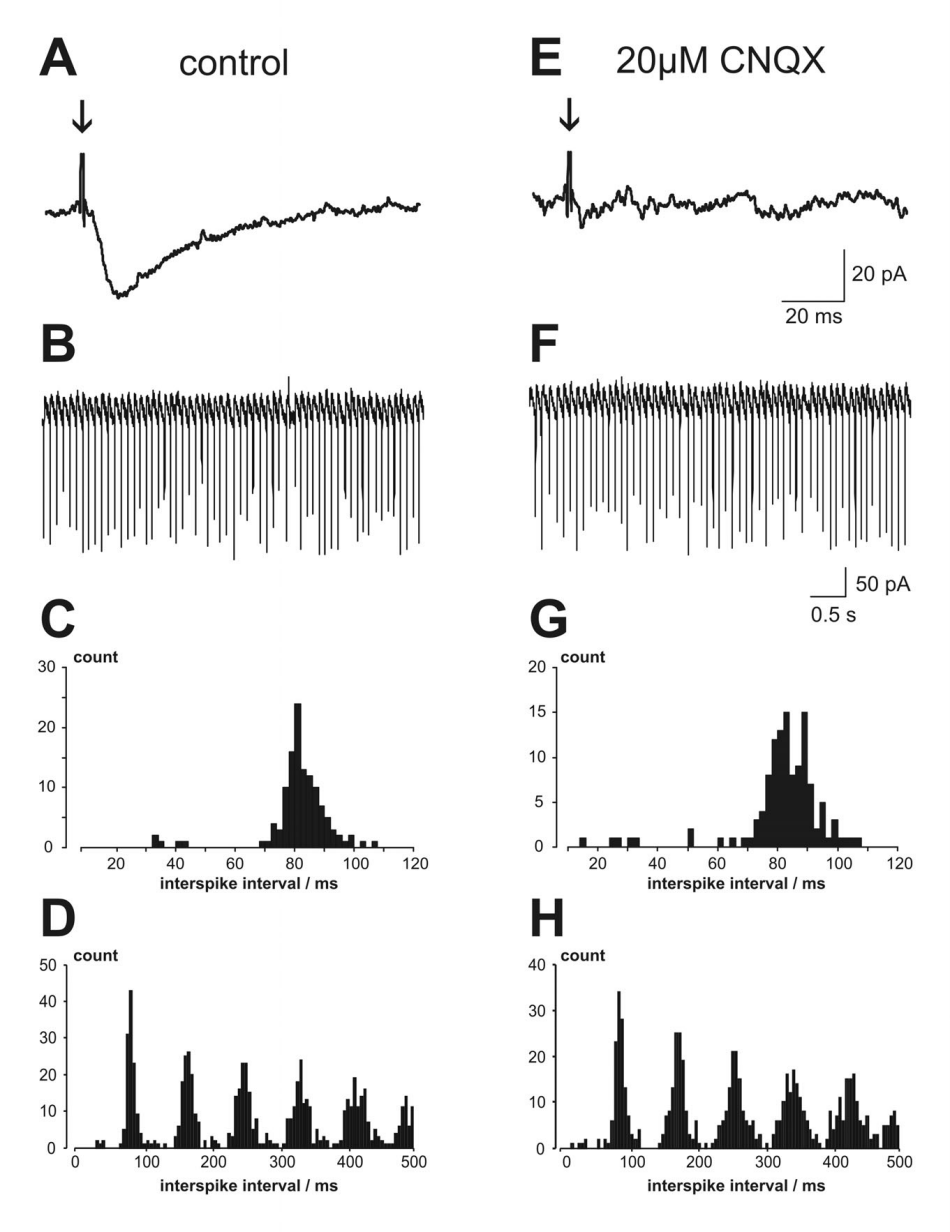
Spontaneous activity of PNC is independent from tonic excitatory input. A single electric shock delivered to the optic tract lateral from the recorded neuron evokes a single peak EPSC (*A*) that completely disappears after AMPA receptor blockade by bath application of 20 μM CNQX (*E*). In contrast, the spontaneous firing rate recorded in control situation (*B*) does not change after AMPA receptor blockade (*F*). Removing excitatory input does also not change the regularity of the firing as derived from the distribution of interspike intervals (*C *vs. *G*) or from the autocorrelogram (*D *vs. *H*).

In order to test whether the spontaneous activity of PNC neurons *in vitro *depends on excitatory input, we first suppressed glutamatergic synaptic transmission and pharmacologically blocked AMPA receptors in 13 spontaneously active PNC cells (Fig. 7). As a control for the effectiveness of AMPA receptor blockade, the influence of the AMPA receptor antagonist CNQX on postsynaptic responses was monitored. In all cells tested, bath application of 20 μM CNQX resulted in a complete loss of EPSCs after electrical stimulation of optic tract afferent fibers (Fig. 7A,7E). Although excitatory input was obviously blocked by CNQX application, the maintained firing remained unchanged (Fig. 7B,7F). In particular, no drop in the firing rate was observed that could have been induced by a possible loss of excitatory input. Furthermore, the comparison of both the ISI distribution (Fig. 7C,7G) and the autocorrelograms (Fig. 7D,7H) obtained from spike trains before and during CNQX application did not show any significant difference. Hence, both the generation of spontaneous activity and its patterning seem to be independent from excitatory input via AMPA receptors. Similar results were achieved when NMDA receptors were blocked by bath application of 50 μM APV or when 2 mM kynurenic acid was applied to simultaneously block AMPA and NMDA receptors (N = 19).

**Figure 8:**
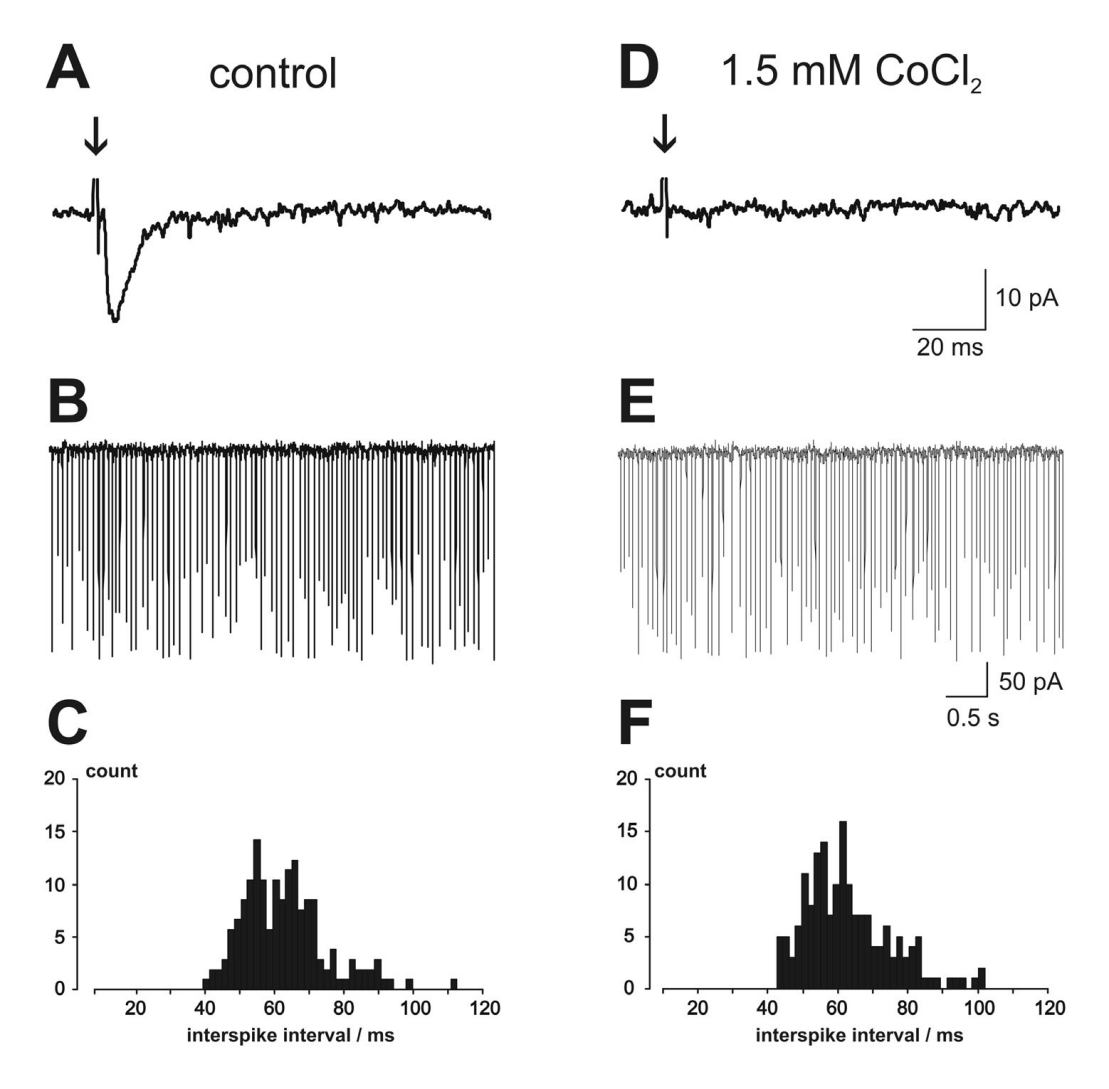
Tonic synaptic input does not contribute to the generation of spontaneous activity in PNC cells. Postsynaptic responses obtained by electrical optic tract stimulation (*A*) can also be blocked by bath application of 1.5 mM CoCl_2 _(*D*). However, the spontaneous firing rate recorded in control situation (*B*) does not change after removal of synaptic input (*E*). Removing excitatory input does also not change the regularity of the firing as derived from the distribution of interspike intervals (*C *vs. *F*).

After having excluded glutamatergic synaptic inputs as a trigger for maintained firing, we tried to remove all synaptic input by adding cobalt to the extracellular medium in 12 spontaneously active PNC cells. This blocks the influx of calcium into the presynaptic terminal and thus prevents vesicular neurotransmitter release. Adding 1.5 mM CoCl_2 _to the bath completely suppressed all electrically evoked postsynaptic currents (Fig. 8A,8D) in all cells tested. In contrast to the complete loss of postsynaptic currents, however, the maintained firing always remained unchanged (Fig. 8B,8E). As during glutamate receptor blockade, no reduction of the firing rate was observed that could have indicated the removal of an excitatory input. In addition, no increase of the firing rate appeared that could have indicated a loss in tonic inhibitory input regulating maintained activity. Finally, examination of the ISI distribution in the spike trains demonstrated that the patterning of the maintained activity also did not show any significant difference in the presence of Cobalt (Fig. 8C,8F). This indicates that spontaneously active PNC cells generate their firing intrinsically without any external synaptic input.

**Figure 9:**
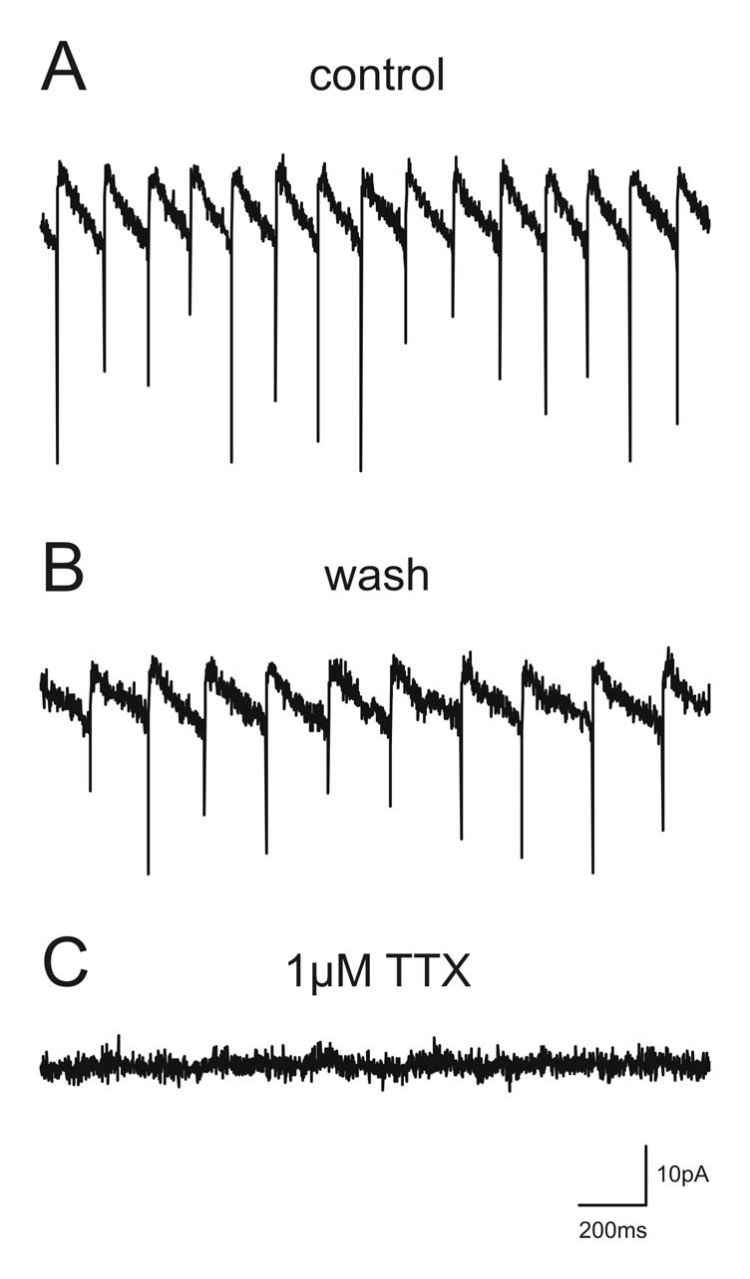
Spontaneous activity in PNC cells depends on a sodium conductance. In control solution (*A*) this cell showed regular firing pattern with depolarizing inward currents preceding each action potential. When 1 μM TTX was added to the bath the firing rate dropped at the beginning of TTX application (*B*). In the presence of TTX, both action potentials and depolarizing inward currents completely disappeared 5 min later (*C*).

In current-clamp mode, each action potential was preceded by a depolarizing ramp (see, for example, Figs. 3C and 4). When cells were hyperpolarized to membrane potentials just below their resting potential single depolarizing ramps appeared that were not followed by an action potential. Concomitantly, in voltage-clamp mode, each unclamped action potential was preceded by an depolarizing inward current (Fig. 9A). Because they did not disappear after substitution of calcium by cobalt in the external solution these current ramps were calcium independent. However, when 1 μM tetrodotoxin (TTX), a selective blocker of sodium channels, was added to the bath solution current ramps were eliminated together with the action potentials (Fig. 9B,9C) in all seven cells tested.

## Discussion

We have examined neurons in the rat PNC that are characterized by maintained activity *in vitro*. These spontaneously active PNC cells do not differ in their dendritic morphology from PNC cells that are not spontaneously active, but they show higher input resistances, more positive resting potentials, and lower spike thresholds. Furthermore, our results indicate that, firstly, all PNC cells that display spontaneous activity share firing characteristics, such as very regularly patterned spike trains and pure tonic firing rate increases in response to intracellular depolarizations. Secondly, the generation of the maintained firing of these cells is independent from excitatory synaptic input which suggests that these cells exhibit specific intrinsic properties that underly the generation of spontaneous activity. Finally, the patterning of the maintained firing is also independent from synaptic input, both excitatory and inhibitory, which indicates that their intrinsic membrane properties determine the firing pattern. To our knowledge, this is the first demonstration of spontaneous activity generated *in vitro *by cells in a subcortical visual relay structure.

### Generation of spontaneous activity *in vitro*

Neurons that show spontaneous activity *in vitro *have been reported to exist in various mammalian CNS structures. Most extensively studied, spontaneously active neurons exist in the suprachiasmatic nucleus (SCN) which contains the biological clock that generates circadian rhythmicity. Thus, SCN cells not only generate spontaneous activity *in vitro *but they also maintain their circadian firing pattern [37-39]. Other populations of cells spontaneously active *in vitro *are the cholinergic interneurons in the neostriatum [40], dopaminergic cells in the substantia nigra [41,42], neurons in the subthalamic nucleus [43,44], neurons in the medial vestibular nucleus [45-47], cells from deep cerebellar nuclei [48,49], and cerebellar Purkinje cells [48,50,51]. Within the visual system, particularly the subcortical portion, spontaneous activity *in vitro *has been described to occur in thalamocortical neurons [52] and in isolated dopanimergic cells from the retina [53]. However, in the mammalian PNC cells that show maintained activity *in vitro *have not yet been reported. These cells are characterized by very regular firing pattern and monotonically increasing firing rates in response to intracellular depolarization. They differed from PNC neurons not spontaneously active by higher input resistance and more positive resting membrane potentials. Because glutamate receptor blockade did not change the firing characteristics, neither the firing rate nor the patterning of the firing, excitatory synaptic input through glutamate receptors seems unnecessary for the generation of the spontaneous activity. Furthermore, maintaining spontaneous firing in these PNC neurons also seems to be independent from synaptic input through other neurotransmitter systems because blockade of synaptic transmission by bath application of Cobalt did not change the firing. Thus, we conclude that the firing pattern is neither shaped by tonic excitation nor by tonic inhibition. Similar to spontaneously active cells in the structures noted above, our PNC neurons must possess intrinsic membrane properties that allow the generation of maintained activity.

As far as the ionic mechanisms underlying the generation of spontaneous activity are concerned our results suggest that it critically depends on a TTX-sensitive sodium conductance. This sodium conductance leads to a steady inward current following spike afterhyperpolarization which induces membrane depolarization to spike threshold. This is similar to the ionic mechanism that is responsible for spike generation in spontaneously active neurons in the suprachiasmatic nucleus [54]. Because spontaneous firing in PNC neurons was unchanged by calcium substitution with cobalt the spontaneous activity generation seems independent from calcium conductances.

### Possible functional implications

A characteristic response property of all spontaneously active PNC cells was that the firing rate increases to depolarizing voltage steps did not show any phasic components. This makes these cells perfectly suited to code maintained or tonic neuronal information. Of course, one has to keep in mind that neuronal response properties *in vivo *are shaped by numeous afferent input systems most of which are absent in the slice preparation. Thus, tonic inhibitory input could mask the maintained firing of spontaneously active PNC cells leading to a very different response pattern *in vivo*. Consequently, the maintained firing might become apparent only under very specific stimulus conditions upon withdrawal of the inhibitory input. However, from a functionl point of view, we regard it more reasonable to assume that PNC cells which are spontaneously active *in vitro *also exhibit tonic firing *in vivo.*

Reviewing the known functions served by PNC neurons *in vivo *reveals only few reasonable suggestions for the possible functions which spontaneously active cells might accomplish. Thus, cells that typically show sustained activity *in vivo *are involved in the pupillary light reflex [2,3,6,26,55]. These cells are characterized by tonic increases of their firing rate to increases in the background luminance. However, these cells are predominantly found in the olivary pretectal nucleus (OPN) which is located in the rostro-medial PNC [reviewed in [56]]. Because our recordings were topographically restricted to the caudo-lateral PNC, particularly to the nucleus of the optic tract (NOT) and the posterior pretectal nucleus (PPN), it seems unlikely that we recorded from luminance neurons in the OPN.

Neurons found in NOT and PPN include various functional cell populations. One of them has been associated with the generation of slow phase eye movements during OKR while others seem to transfer visual information linked to the execution of saccadic eye movements. Cells from these latter populations are all characterized by short duration, high frequency burst responses to fast image motions or rapid eye movements [15,17,25,34-36,57] and thus seem unlikely to correlate with cells that show maintained activity *in vitro*. Furthermore, because the timing of postsynaptic spikes with respect to their presynaptic input might be of considerable functional importance for saccade-related neurons such cells should exhibit lower input resistance than neurons for which spike time precision is less important. Low input resistances allow faster depolarization of the postsynaptic membrane and, hence, less temporal variance or "jitter" between presynaptic and postsynaptic spikes will occur. However, spontaneously active PNC cells on average showed higher input resistances in our sample and we therefore do not think that they represent saccade-related PNC neurons.

On the other hand, cells that control compensatory eye movements during OKN are characterized by tonic firing *in vivo *when appropriately stimulated by low speed horizontal movements of whole field visual stimuli. In all mammals studied, neurons in the right PNC are excited by rightward stimulus motion and control eye movements to the right, while neurons in the left PNC are activated by leftward stimulus motion and control eye movements to the left [8,27-33]. The response properties of OKN-related PNC neurons to a large extent reflect response characteristics of their retinal afferents which are also activated by slow stimulus movements and show strong directional selectivity [58]. However, it may be functionally important to assure a constant level of maintained activity in OKN-related PNC neurons by additional intrinsic mechanisms in the absence of appropriate visual stimuli. Unilateral inactivation of PNC neuronal activity by focal injections of muscimol or lidocaine leads to spontaneous eye movements in darkness [11,59]. Because of the directional specificity in the PNC, inactivation of the right PNC elicits eye movements to the left, while inactivation of the left PNC elicits eye movements to the right. Thus, eye movements that appear after PNC inactivation seem to result from a distortion of a balanced activity between the two PNCs. Whenever the balance is distorted, premotor target structures postsynaptic to OKR-related PNC neurons receive stronger input from the PNC of one side and eye movements are elicited accordingly. It is therefore reasonable to assume that maintained activity spontaneously generated by OKR-related PNC assures this activity balance which is necessary for normal oculomotor function. In order to verify our hypothesis that OKR-related PNC neurons generate spontaneous activity that can be observed *in vitro*, it will be necessary to identify the postsynaptic targets of the spontaneously active PNC neurons.

## Conclusions

We have been able to demonstrate a specific population of neurons in the PNC that is capable of generating spontaneous activity *in vitro*. The spontaneous firing depends on a sodium conductance and is independent from afferent synaptic input. Although the postsynaptic target and, consequently, the functional role of the spontaneously active PNC cells remain to be determined it is reasonable to assume that these cells also show spontaneous activity *in vivo*. Therefore, one likely candidate to represent spontaneously active cells *in vivo *are PNC neurons that are involved in the generation of slow compensatory eye movements during optokinetic nystagmus. If this is true, spontaneous firing might help to maintain an activity balance between neurons in the right and in the left PNC and thus stabilize eye position in the absence of retinal image motion.

## Methods

### Slice preparation

Acute brain slices were obtained from 3 to 6 week-old Long-Evans hooded rats of either sex that had been raised at the institute's own colony. All experimental procedures were in strict compliance with governmental regulations and in accordance with the *Guidelines for the Use of Animals in Neuroscience Research *of the Society for Neuroscience. Animals were deeply anesthetized with halothane and a subcutaneous injection of ketamine (100 mg/kg body weight) and thiazine hydrochloride (1 mg/kg), and transcardially perfused with ice-cold artificial cerebro-spinal fluid (ACSF) containing (in mM), NaCl 123, KCl 2.5, NaH_2_PO_4 _1, NaHCO_3 _26, MgSO_4 _1.3, CaCl_2 _1.8, glucose 11, that was continuously gassed with 5% CO_2 _/ 95% O_2_. After the brain had been removed from the skull, 350 μ m-thick coronal slices were cut on a vibratome in ice cold ACSF. Three to four single slices that included the caudal PNC were obtained from each experimental animal. Slices were kept in ACSF at 36°C for at least one hour to allow recovery from the slicing procedure. For recording, they were transferred to a submerged type recording chamber where they were superfused at 3 ml/min with ACSF at 34°C during patch clamp experiments.

### Whole-cell patch clamp

Whole-cell recordings from neurons in the caudo-lateral PNC were performed under visual guidance using infrared differential interference videomicroscopy [60]. For recording, borosilicate micropipettes (impedance 5–8 MΩ) were filled with internal solution composed of (in mM) potassium gluconate 130, sodium gluconate 5, HEPES 20, MgCl_2 _4, Na_2_ATP 4, Na_3_GTP 0.4, EGTA 0.5, to which 0.5% biocytin was added for morphological single cell reconstruction. Measured membrane potentials were corrected for the junction potential of -10 mV.

Postsynaptic responses were evoked with a concentric bipolar stimulation electrode (SNEX-100X, Rhodes Medical Instruments, Tujunga, CA) placed in the optic tract (OT) at the lateral PNC border. Electrical stimuli delivered were 0.5 to 2 mA in amplitude and had a duration of 100 to 500 μs. The neuronal signals were amplified and filtered using an EPC9 amplifier (Heka, Lambrecht, Germany), digitized at 20 kHz, and displayed, stored, and analyzed using PULSE/PULSEFIT software (Heka, Lambrecht, Germany). Unless otherwise stated, postsynaptic current responses evoked by OT stimuli were averaged over three consecutive stimulus applications. All drug effects are given as mean values ± standard deviation, they were statistically tested for significance using the Student's t-test.

### Drug delivery

All drugs used were obtained from Sigma-Aldrich (Deisenhofen, Germany) and were bath applied. A10-minute application time proved sufficient to achieve stable responses. Application of 20 μM 6-Cyano-7-nitroquinoxaline-2,3-dione (CNQX) was used to block AMPA receptors. Either 50 μM APV or 2 mM kynurenic acid were used to block NMDA receptors. Na currents were suppressed by application of 1 μM tetrodotoxin (TTX).

### Histochemistry

At the end of each recording session, slices were immersion fixed in 4% parafomaldehyde in 0.1 M phosphate buffer, pH 7.4, at 4°C. After at least 24 h in fixative, slices were processed using standard histochemical techniques for visualization of biocytin with 3,3-diaminobenzidine (Sigma-Aldrich, Deisenhofen, Germany). Morphological reconstruction of stained cells was done with the aid of a camera lucida.

## Authors' contributions

NP participated in the design of the study and executed all aspects including data collection, analysis and drafting the manuscript. MS initially designed the study, assisted with data collection and analysis, and edited the manuscript. Both authors read and approved the final manuscript.
